# Use of an electronic metabolic monitoring form in a mental health service – a retrospective file audit

**DOI:** 10.1186/s12888-016-0814-9

**Published:** 2016-04-19

**Authors:** Brenda Happell, Chris Platania-Phung, Cadeyrn J. Gaskin, Robert Stanton

**Affiliations:** Synergy, Nursing and Midwifery Research Centre University of Canberra, Faculty of Health and ACT Health, Canberra, Australia; School of Medical and Applied Sciences, Central Queensland University, Bruce Highway, North Rockhampton, QLD 4702 Australia; Gaskin Research, Melbourne, Australia

**Keywords:** Cardiometabolic, Mental health service, Monitoring, Physical health, Severe mental illness

## Abstract

**Background:**

People with severe mental illness have poorer physical health, experience disparities in physical health care, and lead significantly shorter lives, compared to the general population. Routine metabolic monitoring is proposed as a method of identifying risk factors for metabolic abnormalities. Efforts to date suggest routine metabolic monitoring is both incomplete and ad-hoc, however. This present study reports on the recent implementation of a routine metabolic monitoring form at a mental health service in regional Australia.

**Methods:**

A retrospective file audit was undertaken on 721 consumers with electronic health records at the mental health service. Descriptive statistics were used to report the frequency of use of the metabolic monitoring form and the range of metabolic parameters that had been recorded.

**Results:**

Consumers had an average age of 41.4 years (*SD* = 14.6), over half were male (58.4 %), and the most common psychiatric diagnosis was schizophrenia (42.3 %). The metabolic monitoring forms of 36 % of consumers contained data. Measurements were most commonly recorded for weight (87.4 % of forms), height (85.4 %), blood pressure (83.5 %), and body mass index (73.6 %). Data were less frequently recorded for lipids (cholesterol, 56.3 %; low density lipoprotein, 48.7 %; high density lipoprotein, 51.7 %; triglycerides, 55.2 %), liver function (alanine aminotransferase, 66.3 %; aspartate aminotransferase, 65.5 %; gamma-glutamyl transpeptidase, 64.8 %), renal function (urea, 66.3 %; creatinine, 65.9 %), fasting blood glucose (60.2 %), and waist circumference (54.4 %).

**Conclusions:**

The metabolic monitoring forms in consumer electronic health records are not utilised in a manner that maximises their potential. The extent of the missing data suggests that the metabolic health of most consumers may not have been adequately monitored. Addressing the possible reasons for the low completion rate has the potential to improve the provision of physical health care for people with mental illness.

## Background

People with severe mental illnesses (SMI) -such as schizophrenia, bipolar disorder, and major depressive disorder - have substantially poorer physical health than the general population [1, 2]. They are at increased risk of many physical health problems, including cardiovascular diseases, obesity, metabolic syndrome, and dyslipidaemia [1]. As a result, people with SMI have a reduced life expectancy of between 1.4 and 32 years (median = 10.1 years) [3]. Several lifestyle behaviours (e.g., unhealthy diets, physical inactivity, smoking, alcohol and drug use, and unsafe sex) are known to be contribute to this reduction in life expectancy [4]. In addition, anti-psychotic medication and other psychotropic drugs are known to be major contributors to the ill-health of people with SMI [5–9]. Many factors contributing to the increased risk of cardiovascular and all-cause mortality in people with SMI are modifiable, however [10]. Health care providers, including mental health services, have an important role in identifying and monitoring these risks [11].

The provision of inadequate health care for people with SMI is a major form of inequality [12, 13]. Monitoring of risk factors for poor cardiometabolic health (known as metabolic monitoring) would represent a significant step towards equity in health care provision [14]. As in the general populations, metabolic monitoring in people with SMI is necessary to quantify the risk of cardiovascular disease and diabetes, and initiate early interventions for risk management [15].

Internationally, the level of comprehensive metabolic care offered to people with mental illness is consistently found to be inadequate [16]. Research included in this review comparing the quality of care of people with and without mental illness, showed that people with mental illness taking anti-psychotic medications were 30 % less likely to have their weight, blood glucose, and lipids assessed [16]. Using chart reviews, clinical and administrative data, Littman et al. [17] estimated rates of screening for obesity (defined by body mass index) to be between 94 – 94.7 % in a sample of 37,889 veterans from the Veterans Health Administration in the USA. However, when multiple metabolic risks are considered, monitoring performance is quite variable. Consistent with this evidence, the authors of a recent review reported substantial variability in the rates cardiovascular health screening in people with SMI, which the authors suggested were due to differences in the organization of health care [18]. In a recent study, Cotes et al. [19] inspected medical records within a public-funded community health service in a US state, to ascertain if an education program on the metabolic monitoring of consumers taking antipsychotics would improve monitoring levels. They found a statistically non-significant increase in the frequency of metabolic monitoring, even though the program included feedback on performance at monitoring metabolic risks. Similarly, Saloojee et al. [20] identified that less than one per cent of outpatients at a large South African hospital were screened on the set of risk factors needed to identify metabolic syndrome. Poor monitoring was occurring despite the availability of guideline recommendations on metabolic care of people with SMI in South Africa. Collectively, these studies confirm the variability of metabolic monitoring practices in several countries.

In considering the poor rate of implementation of metabolic care activities, many health care system-related factors (e.g., lack of role clarity within treating teams regarding responsibility for screening, lack of continuity of care, limited time and resources for physical and medical examinations, separation of medical and mental health care systems) represent significant barriers to change [21, 22]. Moreover, nurses working in mental health have reported personal barriers to monitoring, such as competing priorities and concerns regarding the outcomes of screening [23]. The increased metabolic risks for people with SMI are well documented [24], particularly for those consumers prescribed anti-psychotic medication [1, 9]. Therefore, it is fundamental to clinical practice to have routine and detailed metabolic monitoring to track changes in risk to inform clinical decision-making.

There is limited data on metabolic monitoring rates in Australian mental health care services, with reports primarily available from metropolitan, rather than regional and rural, areas [25] e.g. [26]. Organ et al. [27] reported that around 60 % of consumers files contained data on lipid and blood glucose levels, whereas only 7 % of consumers with SMI had their weight circumference recorded. The absence of waist circumference measurement is also reported in other studies. For example, Rosenbaum and colleagues [28] reported that, prior to an educational intervention for nurses highlighting the importance of waist circumference assessment, these data were not reported in any of the 60 randomly sampled consumer files selected for auditing. Three months later, 58 % of files contained waist circumference measurements, while at nine months this reduced to 42 %. Although barriers to waist circumference measurement (such as concern regarding physical contact) exist, the study of Rosenbaum and colleagues [28] demonstrated that educational interventions for nurses can improve the assessment of cardiometabolic markers in people with SMI.

Thompson et al. [29] reported metabolic monitoring levels of consumers with first episode psychosis on a regimen of anti-psychotic medications in a service adopting locally adapted monitoring guidelines. Blood pressure data was reported in 41.6 % of files reviewed, blood glucose data was reported in 24.4 % of cases, while lipids were reported in 26.7 % of files. In the same 86 consumers tracked over six months, initial recordings (screening stage) were higher – 81.4 %, 74.4 % and 75.6 % respectively - indicating efforts are needed to sustain attention on metabolic risks.

To combat the poor physical health care of people with SMI, specialist nursing roles have been proposed. In a recent study, McKenna and colleagues [30] compared rates of metabolic monitoring between a mental health service where such a role was implemented, with a service where no such role existed. A specialist role led to a significant increase in the rate of metabolic monitoring, compared to services where this responsibility fell to case managers. Despite this specialist role initiating metabolic monitoring in 78 % of first episode consumers, there were metabolic monitoring data missing in more than 42 % of cases, with smoking the only parameter monitored in all cases. In reviewing the reasons for missing data, McKenna and colleagues note that specialist nursing staff identified several reasons for the missing data, including consumer anxiety towards needles (limiting the potential to undertake blood profiling), the complexity of procedures for collecting fasting blood glucose, logistical barriers to attending community-based pathology services (e.g., timing of appointments or transportation issues), and poor communication of diagnostic results between the mental health service and local medical officers.

Collectively, these studies highlight the poor use of metabolic monitoring in metropolitan mental health services. Such findings may well generalise to regional services. This paper reports on metabolic monitoring in a regionally located public mental health service within a large state of Australia. In mid-2013, the health service implemented a policy of routine metabolic monitoring of all consumers using antipsychotic medications or mood stabilisers. A standardised metabolic monitoring form was developed to be completed and uploaded to the electronic medical records database of consumers accessing the service. Given the high prevalence of cardiometabolic risk factors, and the repeated calls for routine metabolic monitoring in this population [31], the introduction of a standardised metabolic monitoring form represents a significant step in addressing the poor physical health of people with mental illness. However, the effectiveness of such a strategy remains to be evaluated, particularly in regional areas. Therefore the aims of the present study were to evaluate the frequency of use of the routine metabolic monitoring form and to examine the degree to which the forms were completed. Such data are important in evaluating the implementation of routine metabolic monitoring strategies, and the outcomes have important implications for mental health service delivery.

## Methods

### Setting

This study was undertaken in a mental health service in a city of approximately 75,000 people, located in regional Queensland, Australia. The service provides both inpatient and community mental health services and receives approximately 85 referrals per month.

### Design

A retrospective file audit was undertaken examining the use of the metabolic monitoring form in the regional mental health service. Where multiple recording of metabolic monitoring data were contained on a single form for a consumer, the earliest data was taken as baseline data, with subsequent entries taken as the follow up time points.

### Data

The files of 721 consumers were reviewed. The consumers’ ages ranged from 13 to 83 years (*M* = 41.4 years, *SD* = 14.6), over half were male (58.4 %), and the most common psychiatric diagnosis was schizophrenia (42.3 %) (see Table 1).

**Table 1:** Demographic and psychiatric diagnostic differences between consumers for whom the metabolic monitoring form had and had not been used

	Total sample (*n* = 721)	Form used (*n* = 261)	Form not used (*n* = 460)	Effect size	*p* value
Age, mean (SD) years	41.4 (14.6)	41.4 (14.3)	41.5 (14.8)	*d* = 0.01	.902
Sex				φ = .12	.001
Males, *n* (%)	421 (58.4)	175 (41.6)	246 (58.4)		
Females, *n* (%)	300 (41.6)	89 (29.7)	211 (70.3)		
Diagnosis (ICD-10)^a^				Cramer’s *V* = .27	<.001
Schizophrenia (F20), *n* (%)	305 (42.3)	159 (60.7)	146 (33.1)		
Bipolar disorder (F31), *n* (%)	84 (11.7)	23 (27.4)	61 (72.6)		
Depressive disorders (F32, F33), *n* (%)	81 (11.2)	17 (21.0)	64 (79.0)		
Anxiety disorders (F41), *n* (%)	40 (5.5)	7 (17.5)	33 (82.5)		
Other, *n* (%)	193 (26.8)	56 (29.0)	137 (71.0)		

^a^Diagnosis data were missing for 18 (2.5 %) consumers

*SD* standard deviation; ICD- 10

### Metabolic monitoring form

As there is no consensus or minimum data set recommendation for metabolic monitoring forms, the form developed and implemented in this service was based on the needs of the service in consultation with clinicians. This form enables the recording of metabolic-related data, including anthropometrics (e.g., waist circumference, weight), blood markers of cardiovascular and endocrine health (e.g. triglycerides, alanine aminotransferase [ALT]), medication specific blood markers (e.g., valproate level, thyroid function test results, lithium levels), and electrocardiogram findings (e.g., rate, rhythm, abnormality). Data recorded on the form are uploaded to consumers’ electronic health records.

### Procedures

The data collection occurred between November 2014 and April 2015 and included all consumer electronic health records at the service. A trained research assistant extracted all data from consumers’ metabolic monitoring forms to a spreadsheet. All data were de-identified and only data that appeared on the metabolic monitoring form was extracted.

### Statistical analysis

To determine whether there were differences in the ages, sexes, and psychiatric diagnoses of consumers for whom the metabolic monitoring form had, and had not been used, appropriate inferential statistics (i.e., a paired *t* test for age and *χ*^2^ tests for sex and psychiatric diagnoses) were applied. Consistent with sound statistical practices [32, 33], effect sizes were calculated and the issue of experiment-wise alpha inflation due to multiple tests was addressed. Effect sizes are reported in the form of Cohen’s *d*, φ, and Cramer’s *V*. According to Cohen’s [34] conventions, small, medium, and large effects are 0.2, 0.5, and 0.8, respectively, for Cohen’s *d* and .10, .30, and .50, respectively, for both φ and Cramer’s *V*. With only three tests performed, setting alpha at .01 adequately addresses the problem of inflated experiment-wise error due to multiple testing. To assess the extent to which the form was being completed, descriptive statistics (*n*, %) are reported.

### Ethics

Ethical clearance for the conduct of this retrospective file audit was granted by the health service (Queensland Health) and institutional (CQUniversity) human research ethics committees prior to the commencement of this study. Health service approval was granted to access data contained in consumer files.

## Results

Metabolic monitoring forms were available for 261 (36 %) consumers. Comparing consumers where forms were available, with those were the form was not available, there were no differences in ages, but forms were present for higher percentages of males, and for consumers with schizophrenia (see Table 1). For the 261 consumers for whom baseline data were available, 59 (22.6 %) had follow up data recorded in their forms at three months, 87 (33.3 %) at six months, 42 (16.1 %) at 12 months, 18 (6.9 %) at 18 months, 9 (3.4 %) at 24 months, and 1 (0.4 %) at 36 months.

Data were not recorded for many of the measures included in the form. Although height, weight, and blood pressure were each recorded for over 80 % of consumers, other data applicable to all consumers were recorded in half to two-thirds of forms (see Table 2). With respect to medication-specific measures, data were recorded in between 1.1 % and 20.7 % of forms. Data relating to cardiac function were never recorded (see Table 3).

**Table 2:** Data recorded in the metabolic monitoring form at baseline

	Measure	Observation recorded – *n* (%)	Observation not recorded – *n* (%)
Anthropometry	Height	223 (85.4)	38 (14.6)
	Weight	228 (87.4)	33 (12.6)
	Body mass index	192 (73.6)	69 (26.4)
	Waist circumference	142 (54.4)	119 (45.6)
Blood pressure	Blood pressure	218 (83.5)	43 (16.5)
Fasting blood glucose	Fasting blood glucose	157 (60.2)	104 (39.8)
Lipids	Cholesterol	147 (56.3)	114 (43.7)
	Low density lipoprotein	127 (48.7)	134 (51.3)
	High density lipoprotein	153 (51.7)	126 (48.3)
	Triglycerides	144 (55.2)	117 (44.8)
Liver function	Alanine aminotransferase	173 (66.3)	88 (33.7)
	Aspartate aminotransferase	171 (65.5)	90 (34.5)
	Gamma-glutamyl transpeptidase	169 (64.8)	92 (35.2)
Renal function	Urea	173 (66.3)	88 (33.7)
	Creatinine	172 (65.9)	89 (34.1)

**Table 3:** Medication-specific observations recorded in the metabolic monitoring forms at baseline

	Measure^a^	Observation recorded – *n* (%)	Observation not recorded – *n* (%)
Endocrine	Prolactin level^b^	27 (10.3)	234 (89.7)
Sodium valproate	Full blood count	8 (3.1)	253 (96.9)
	Valproate level	26 (10.0)	235 (90.0)
Carbamazepine	Full blood count	1 (0.4)	260 (99.6)
	Carbamazepine level	3 (1.1)	258 (98.9)
Lithium	Thyroid function test	54 (20.7)	207 (79.3)
	Lithium level	3 (1.1)	258 (98.9)
Clozapine	Clozapine level	40 (15.3)	221 (84.7)

^a^Space is also provided on the form to record observations of cardiac function, including electrocardiogram (rate, rhythm, abnormality), echocardiogram (if consumers are on Clozapine), and troponin. No forms contained observations for these measures

^b^Instructions on form are to record if consumer is on Risperidone or Amisulpride

## Discussion

In the present study, we examined the use of an electronic metabolic monitoring form in a mental health service in regional Queensland, Australia. Few studies have examined this aspect of mental health care, and those published to date have similar findings to ours. For example, Organ and colleges [27] audited 618 files from a large metropolitan mental health service and reported that there had been inconsistent recording of cholesterol (63 % of files), blood glucose (60 % of files), body weight (54 % of files) and blood pressure (44 % of files). Waist circumference data was present in only 7 % of files. More recently, Rosenbaum and colleagues [28] reported that blood pressure was recorded on 92 % of files and body mass index was recorded on 78 % of files, whereas waist circumference was not reported on any of the 60 files audited at baseline. Finally, an audit of metabolic monitoring within a large metropolitan community mental health service revealed that files contained data on body weight (61 % of files), blood glucose (59 % of files), lipids (49 % of files), and blood pressure (38 % of files) [35]. In contrast to the aforementioned studies, waist circumference was not included in the metabolic monitoring study described by Millar and colleagues, as this parameter was not assessed by the service. The findings, both from our study and from previous work, highlight both the lack of consensus on the parameters on metabolic monitoring and the extent of missing data on consumers’ physical health. This missing data was especially evident in the recording of data relating to cardiac function, which, although detailed on the form, was not reported in any file audited.

For over a century, the health care inequalities mental health consumers routinely experience with respect to their physical health needs have been consistently documented ([36, 37], e.g., [38–41]). This inadequate attention to the physical health needs of mental health consumers is a likely contributor to the higher than expected mortality rates in this population [42]. Exposing the magnitude and consequences of this problem provides healthcare professionals and service providers with opportunities to gain a clearer understanding of the challenges inherent in the care of mental health consumers and to develop and trial possible solutions. Unfortunately, our research provides further evidence that the problem of poor physical healthcare of mental health consumers has not been adequately addressed.

Given the high prevalence of metabolic abnormalities, such as hypercholesterolaemia, among mental health consumers [43, 44], particularly those with schizophrenia [45], the findings of this study are particularly concerning. Our findings are, however, consistent with previous research showing that metabolic risk factors are not adequately monitored in mental health consumers [8]. Data had been entered into the electronic metabolic monitoring forms for one third of the consumers whose files were accessed. In these forms, substantial data were missing. This finding suggests that inadequate monitoring of metabolic risk factors reported in studies undertaken in mental health services in metropolitan area of Australia [28–30, 46] may be applicable to regionally-located services. Consistent with other studies [27, 28] the absence of waist circumference data in many consumer files was also observed in this study.

Several factors may have contributed to the limited use of the form. Firstly, forms may not have been present in some electronic files because those consumers may not have had contact with the health service since the implementation of the routine metabolic monitoring form. Secondly, although service policy was that the electronic forms were to be used, a period of transitioning from paper-based to electronic forms means that some staff may have continued to use the paper-based forms. Discussions with the health service, however, indicated that some medication-specific observations (e.g., lithium levels) were not routinely conducted or only conducted in specific cases. Thus, it is unclear if the limited use of the form results from observations not being made or data being recorded in paper-based rather than electronic format. Thirdly, some staff may not have been trained in the use of the new initiative or may have chosen not to use the form based on personal preference. Finally, some consumers may have exercised their right to refuse to participate in some or all routine tests. This last point is important and highlights a limitation in the current form in that a consumer’s refusal is not record and this may have contributed to the low reporting of some measures.

There are a number of service-level strategies that may advance the use of electronic metabolic monitoring forms to better manage the physical health care of people with SMI. For example, regular and ongoing audits to manage the transition from paper-based to electronic metabolic monitoring records could be initiated and linked to key performance indicators. These audits should identify additional information of interest, including the pharmacotheraputic outcomes and adverse events, and incorporate electronic flagging of results that are outside reference ranges, to ensure follow up and early intervention. Nurses, for example, with their holistic training, may be well-placed to facilitate a multidisciplinary approach to the mental and physical healthcare of mental health consumers [47]. Notably, a high number of nurses in mental health recognise physical health care as an important part of their role and express interest in ongoing education and training on cardiovascular health and diabetes [48]. Indeed, a number of studies have demonstrated the success of specialist nursing roles for coordinating the physical health care of mental health consumers [23, 26, 30]. Integration of these roles in to routine practice, however, is not widespread. As a result, the ongoing physical health care of mental health consumers in mental health settings remains disjointed.

The findings of the present study are not without limitation. The data for this study were obtained from the metabolic monitoring forms available in consumers’ electronic health records only. Therefore, we have no knowledge of the medications consumers were taking at the time data were entered on the form. As such, we are unable to determine whether metabolic monitoring was more thorough or more frequent for those consumers who were taking medications known to increase cardiometabolic risks. Equally, we cannot determine if pharmacotherapeutic agents for the treatment of hypertension, hyperglycaemia, hypertriglycidaemia or hypoalphalipoprotienaemia were used. This limitation, however, highlights a significant issue with the current metabolic monitoring form. More broadly, the extent of missing data in the metabolic monitoring form suggests that either routine testing was not undertaken for some consumers or the data is reported elsewhere. Future studies in this field should consider the correlates to routine metabolic monitoring and the effect of clinician training on use of the form, to enhance its role in clinical decision-making.

The design of the study (a retrospective file audit) does not facilitate an understanding of the reasons why not all measures were present on the form. In mental health care services potential reasons for not undertaking or reporting data on metabolic risks include time constraints, views that screening can negatively affect rapport building, and absence of equipment [27, 46]. Qualitative research with nurses within mental health care indicate an awareness of the physical health problems experienced by people with SMI [49–51]. However, nurses express concerns regarding physical health care such as competing priorities, role delineation, and access to physical health interventions for consumers [49]. In an interview study that included nurses (*n* = 21), Ehrlich et al. [51] came to an overriding theme of “care boundaries” that nurses needed to navigate, as those boundaries shaped their individual agency to provide physical health care. The boundaries included “the illness (e.g. acuity), care provision processes (e.g. professional scope of practice), sectors (i.e. government, non-government, and private), the health-care system (e.g. funding constraints), and society (e.g. societal norms surrounding mental illness)” ([51], p. 245).

## Conclusions

Inadequate physical health care of people with SMI is acknowledged as a widespread issue [52]. The present study demonstrates that a routine metabolic monitoring form placed in the consumers electronic health record was not utilised in a manner that maximises its potential benefit to the consumer or the health service. A great deal of missing data is evident and interventions or treatments to address metabolic abnormalities are not recorded on the form. Concerted, systematic changes are required within mental health services so that consumers can enjoy their right to equitable healthcare.

### Ethics

Ethical clearance for the conduct of this retrospective file audit was granted by the Human Research Ethics Committees of Queensland Health (HREC/14/QPCH/150) and CQUniversity (H14/08-180) prior to the commencement of this study. Health service approval to access data contained in consumer files was granted under the Queensland Health Public Health Act.

### Consent to publish

Not applicable.

### Availability of data and materials

The Public Health Act approval to access the data used in the preparation of this manuscript does not extend to making it available in the public domain. Therefore, data and materials cannot be shared.

## Abbreviations

ALT: alanine aminotransferase
SMI: severe mental illness
